# Land Use Changes and Their Effects on the Value of Ecosystem Services in the Small Sanjiang Plain in China

**DOI:** 10.1155/2014/752846

**Published:** 2014-03-09

**Authors:** Jing Chen, Bo-Ming Sun, Dan Chen, Xin Wu, Long-Zhu Guo, Gang Wang

**Affiliations:** Key Laboratory of Efficient Irrigation-Drainage and Agricultural Soil-Water Environment in Southern China of Ministry of Education, College of Water Conservancy and Hydropower Engineering, Hohai University, Nanjing 210098, China

## Abstract

The small Sanjiang plain is one of the most important commodity grain production bases and the largest fresh water wetland in China. Due to the rapid expansion of agricultural activities in the past 30 years, the contradiction between economic development and the loss of ecosystem services has become an issue of increasing concern in the area. In this study, we analysed land use changes and the loss of ecosystem services value caused by these changes. We found that cropland sprawl was predominant and occurred in forest, wetland, and grassland areas in the small Sanjiang plain from 1980 to 2010. Using a model to evaluate ecosystem services value, we calculated that the decreased values of ecosystem services were 169.88 × 10^8^ Yuan from 1980 to 2000 and 120.00 × 10^8^ Yuan from 2000 to 2010. All of the ecosystem services were diminished from 1980 to 2010 except for food production. Therefore, the loss of ecosystem services value should be considered by the policymakers of land use and development.

## 1. Introduction

Natural ecosystem services are those products and functions that contribute to humankind and other living organisms' survival while also improving the quality of human life [1]. The ecosystem services include food production, hydrology adjustment, climate regulation, biodiversity maintenance, gas regulation, waste treatment, erosion control, and entertainment [2–5]. The provision of ecosystem services is directly affected by land use [6–8]. However, because of population and economic growth, urban sprawl, and the quick expansion of industrial and agricultural activities, land use has experienced enormous changes worldwide over the past half century, from forest, grassland, and wetland to arable and building land. These changes have caused natural ecosystems to continuously be altered, destroyed, and diminished [4, 6, 9–14]. Because of the increasingly imbalanced provision of economic and natural ecosystems, the effects of land use changes on ecosystem services and their economic value have become a focus of concern for scientists, policymakers, and stakeholders over the last decade [15, 16].

Quantitative assessment of the effects of land use changes on the value of ecosystem services is one of the research focuses of sustainable development in science [17, 18]. Many scholars have conducted studies in different countries, regions, and basins since the 1990s [19, 20]. The effects of coastal erosion on the value of ecosystem services in Europe were quantitatively evaluated with the result that the loss of ecosystem service values was a *€*23 million decrease from 1975 to 2006 [21]. In Chachalacas of Mexico, due to increased urban sprawl and the decrease of grasslands and croplands from 1995 to 2006, the net loss of ecosystem service value ($US 2006/ha/year) was approximately $7 × 10^5^ [4]. Rapid land use changes in Uruguay over the past 20 years, from grassland to plantations, had seriously affected the provision of ecosystem services [22]. The analysis of land use changes and their consequent changes in ecosystem services value in the Huairou reservoir basin in China showed that in 2008 the ecosystem services value had increased 2.88% compared with that in 1990 [20]. These studies offer theories and explore land use options and the sustainable development of ecosystems in these areas.

The small Sanjiang plain is one of the most important commodity grain production bases in China. Agriculture is the leading industry in the region, and its major functions are food production and the provision of jobs and income for the local rural population [23]. The expansion of cropland in response to economic needs has been the main driver of agriculture in the region [24, 25]. A rapid expansion of agricultural activities has caused an enormous change in land use in the small Sanjiang plain in the past 30 years, from a mass of forest, grassland, and wetland to farmland [26, 27], resulting in natural ecosystems being altered and destroyed and many ecological problems such as soil erosion, nonpoint source pollution, the decrease of soil fertility, and a decline in biodiversity [28–30]. Currently, the imbalanced provision of economic and ecosystem services that is a main restricted factor in social and economic sustainable development has become an issue of increasing concern in the small Sanjiang plain.

The main goals of this study are to assess land use changes in the small Sanjiang plain from 1980 to 2010 and to evaluate the effects of these changes regarding ecosystem services. We used the value of ecosystem services to estimate the effects of regional land use changes associated with agricultural development that has occurred over the past 30 years (1980–2010).

## 2. Materials and Methods

### 2.1. Study Area

**Figure 1:**
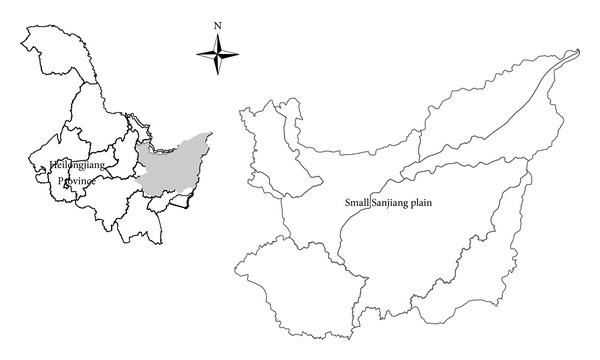
Location of the study area in Heilongjiang Province, northeast China.

The small Sanjiang plain (46°20′40′′*~*48°27′40′′N, 129°11′20′′*~*135°05′26′′E) is located in Heilongjiang Province in northeast China (Figure 1). It is an alluvial plain deposited by the region's three major rivers: Heilongjiang River, Songhuajiang River, and Wusulijiang River. The area of the plain is approximately 6.9 × 10^4^ km^2^ and contains 15 counties and cities. The small Sanjiang plain lies in a temperate, semihumid continental monsoon climate, and the average annual temperature is 1.8°C [29]. Annual precipitation is 532 mm in the plain (falling mainly from July to September) [30]. The average annual land surface evaporation and water surface evaporation (E601 evaporation pan) are 300–500 mm and 550–840 mm, respectively. Because of three large agricultural developments and the rapid expansion of agricultural activities by 2010, farmland area accounted for 62.7% of the plain area [31], including paddy fields (45.8% of the arable land) and dry fields (54.2%). The main crops include rice, soybeans, corn, and wheat.

### 2.2. Land Use

In this study, three Landsat TM images from the years 1980, 2000, and 2010 were selected as the data source for land use. The pretreatment was shown in these images, which contained unified projection system and geometric correction (the error is not more than half a pixel) [32]. Geographical information systems (GIS 10.0) were used to analyse and build up a database of land use. Considering the land use classification system of both China and our study, we divided land use into six classes: (1) farmland (dry land and paddy land), (2) forest, (3) wetland, (4) grassland, (5) building land, and (6) wasteland.

### 2.3. Ecosystem Services Value (ESV)

Due to a desire for more benefits and a lack of knowledge of ecosystem services value, humans have globally developed many natural ecosystems into cropland and building land in the past, resulting in altered and destroyed functions of ecosystem services and the reduced provision of ecosystem goods and services to society [2, 11, 33]. With a more thorough understanding of the fact that ecosystem services cannot be substituted, the study of ecosystem services value has gradually garnered greater attention [20, 34].

Several methods were used to estimate the value of ecosystem services, such as the simulated market approach [35], the surrogate market approach [36], energy system [37], and benefit transfer [2, 38]. The benefit transfer method was used to estimate ecosystem services of global biomes, which was considered to be a classical method by the authors [39, 40]. However, other scholars argued that this method was not a model for the entire world, especially China [3, 20]. The method for estimating ecosystem services value in China was improved by interviewing 800 Chinese ecologists [14]. This method was considered to be more accurate and practicable in estimating the value of ecosystem services in China [18, 34, 40].

In the present study, we used the benefit transfer method to estimate ESV in the Sanjiang plain based on the result of [14]. The ESV for different years can be calculated as follows:

(1) $$\begin{array}{c} {\text{ESV}}_{n}=\underset{k}{\sum }\underset{f}{\sum }A_{nk}\times \text{V}{\text{C}}_{kf}, \end{array}$$

where *A*_*nk*_ is the area of land use for type *k* in *n* year; *n* is 1980, 2000, and 2010; VC_*kf*_ is the value coefficient of ecosystem services value for type *f* (RMB Yuan/hm^2^) (Table 1) with land use type *k*.

Finally, to analyse the impact of land use changes on the value of ecosystem services in two periods (1980–2000, 2000–2010), we calculated the change rate of ecosystem services value yearly. The change rate can be calculated with the formula

(2) $$\begin{array}{c} {ð}_{i-j}={\left(\frac{{\text{ESV}}_{n_{j}}}{{\text{ESV}}_{n_{i}}}\right)}^{1/(j-i)}-1, \end{array}$$

where *ð*_*i*−*j*_ is the rate of change in two periods (1980–2000, 2000–2010); ESV_*ni*_ is the value of ecosystem services in *n*_*i*_ year; *n*_*i*_ is 1980, 2000, and 2010.

## 3. Results

### 3.1. Land Use Change

**Figure 2:**
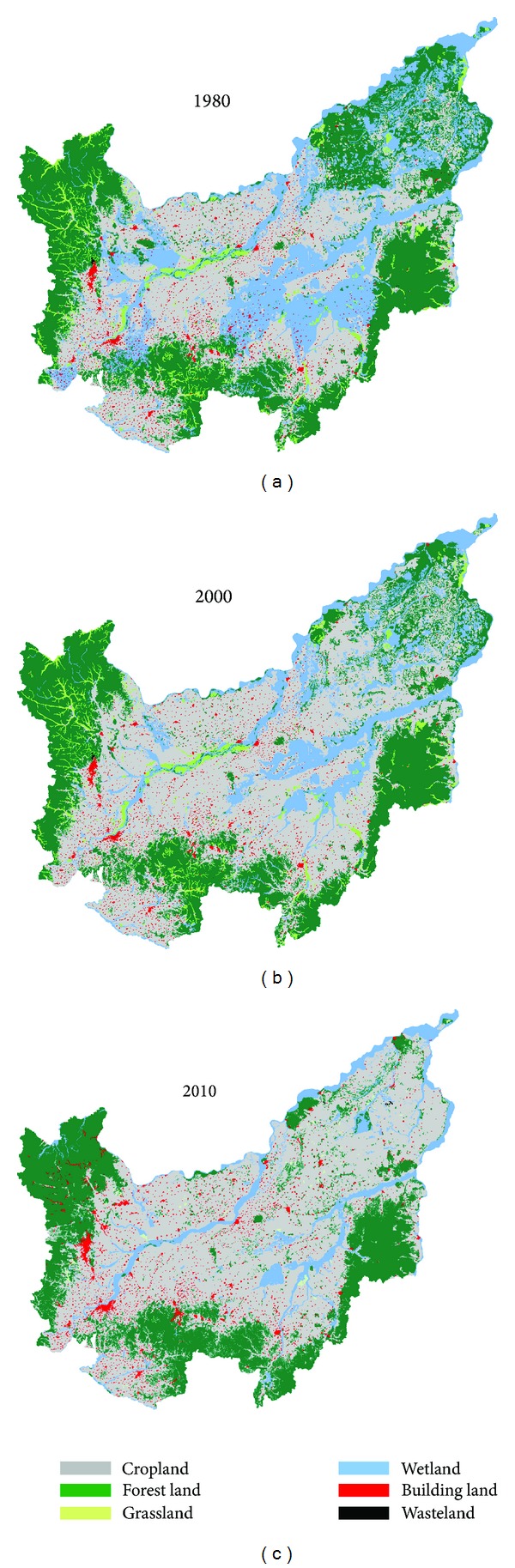
Land use changes over time in the small Sanjiang plain.

Land use changes in different periods and a spatial illustration were calculated by GIS and Office Excel (Figure 2, Table 2). We found that cropland and forest always occupied the largest area in 2010, with the proportion being approximately 89%, followed by wetland, building land, grassland, and wasteland. During the study period, cropland and building land had an increased area, while other land uses decreased.

The increased area of cropland was the largest, measuring at 172.63 × 10^4^ hm^2^ from 1980 to 2010 (Table 2), with an average annual increased rate of 2.2%. Particularly during the last decade, the expansion of cropland was rapid, with an average annual increased rate of approximately 2.5% (the rate of 1.6 from 1980 to 2000). The increased area of building land was 5.3 × 10^4^ hm^2^ from 1980 to 2010. The average annual increased rates of the different periods were approximately 0.3% (1980–2000) and 3.6% (2000–2010).

The trend of wetland changes was the opposite to that of cropland, with its decreased area being the largest (Figure 2, Table 2). The area of wetland decreased from 171.74 × 10^4^ hm^2^ in 1980 to 56.29 × 10^4^ hm^2^ in 2010, with an average annual decreased rate of approximately 2.4%. From 1980 to 2000, the decreased area of wetland was approximately 67.11 × 10^4^ hm^2^. This result shows that the developed wetland had been converted to cropland.

The rate of change of grassland was the largest, with a decreased rate of approximately 92% from 1980 to 2010 (Table 2). Grassland area was approximately 2.1 × 10^4^ hm^2^ in 2010, which accounted for approximately 0.3% of all the plain area. In other words, grassland is currently almost nonexistent in the small Sanjiang plain. The rate of change of forest is the lowest, with the decreased rate being 17.85% from 1980 to 2010.

### 3.2. Ecosystem Services Value (ESV)

In this study, ecosystem services include gas regulation, climate regulation, hydrology adjustment, erosion control, waste treatment, biodiversity, food, raw materials, and entertainment (Table 1). Using the ESV model (formula (1)) and the previously described data, we estimated the ESV change in different periods in the small Sanjiang plain. The results are shown in Tables 3 and 4.

Table 3 shows that the total value of ecosystem services experienced a downtrend. The total ESV in 1980 was 801.57 × 10^8^ Yuan. In comparison, ESV in 2000 and 2010 decreased approximately by 23.4% and 35.4%, respectively. From our own calculations, we found that forest, wetland, and cropland are the main providers for the ecosystem service value. The ESVs of forest and wetland were the largest, however, with their ESVs decreasing from 1980 to 2010. Because of the rapid expansion of agriculture, the increased ESV of cropland was 56.07 × 10^8^ Yuan from 1980 to 2010. In addition, in our study, because the ecosystem services values of building land and wasteland were considered as ESV = 0 [4], although building land and wasteland should had on gain in ESV, but loss. From our calculations (using formula (2)), Table 4 shows that the values of all single ecosystem services decreased from 1980 to 2010, except for food. The values of hydrology adjustment, climate regulation, and waste treatment were the most marked decreases. The reason for this phenomenon was that forest and wetland had been overexploited in the past 30 years. From 2000 to 2010, food production had a huge increase, with an average annual increased rate of 9.91%.

## 4. Conclusion and Discussion

Looking at Figure 2 and Table 2, we observed that land use changed sharply over 30 years in the small Sanjiang plain. For more benefits and a better quality of life, humans developed natural ecosystems into industrial land and cropland in this plain. In the small Sanjiang plain, forest, grassland, and wetland decreased markedly, resulting in losses of ecosystem services and the decrease of ESV, although these changes occurred at varying degrees (Tables 3 and 4). The decreased ESVs of the different periods were 169.88 × 10^8^ Yuan (1980–2000) and 120.00 × 10^8^ Yuan (2000–2010).

Several factors caused these decreasing trends. On the one hand, the decreased area of forest and wetland approximately equalled the increased area of cropland and building land, and yet their ecosystem services values were higher, resulting in the ecosystem services value being decreased. On the other hand, in our study, we cannot quantitatively estimate the ecosystem services value of building land, but its area increased. Furthermore, we find that food output of cropland in Table 2 is undervalued, compared with the average of Heilongjiang Province (3300 kg/hm^2^). Thus, if the food output of farmland increased to 3000 kg/hm^2^, the decreased rate of ESV in different periods will be 155.90 × 10^8^ Yuan (1980–2000) and 94.12 × 10^8^ Yuan (2000–2010).

Because of rapid population growth, the government is currently facing an enormous challenge of securing an adequate food supply in China [41, 42]. The small Sanjiang plain is one of the most important commodity grain production bases, and its food production has an important impact on ensuring food security in China. Thus, for obvious reasons, food production is a significant driver of land use changes in the small Sanjiang plain. Land use has experienced major changes in the past 30 years, especially in the last decade. Because many forests and wetlands are replaced with cropland, the result is an altered and diminished provision of ecosystem services and functions (apart from food production) to society, such as a decline in climate and hydrology adjustment and a decrease in biodiversity decrease, among other effects [30].

The loss of wetland and forest was the most important change in the past 30 years. Because of current climate change scenarios such as an increased frequency of storms and floods [28, 43], it has become more important to protect against such events. The small Sanjiang plain suffered its heaviest floods in 2013, especially in regions where the capacity to retain water is poor. Additionally, the rapid growth of wetland tourism has provided a further reason to preserve wetland and its very high economic and ecological value.

The environmental problems of land use changes are slowly beginning to be considered in the decision-making process regarding land use. For instance, one of the goals of the water conservancy development plan of the Sanjiang plain (2005–2030) was to protect the natural ecological environment and return cropland to wetland and forest. This plan noted that the natural ecosystem that provides goods and services, such as hydrology adjustment, habitat support, and soil fertility, was very important for social sustainable development. However, although the importance of ecosystem services has been acknowledged, the value of ecosystem services has thus far not been considered for any development plans. Therefore, the present study can provide the positive influence and theoretical basis for protecting the natural ecological environment, in which the contradiction is evident between sustainable ecosystem services and land exploitation.

Due to ever-increasing agricultural expansion worldwide (especially in China), land use changes may seem increasingly economically profitable. However, when land use changes deplete the ecosystem's capacities to deliver ecosystem services, long-term losses may exceed short-term gains. Land use and policy making should aim at balancing society's needs and preferences, while considering ecosystem service losses as, in the long-run, it will be beneficial for all of us if natural ecosystems are preserved and used adequately.

## Figures and Tables

**Table 1:** Ecosystem services value per unit area for different land use in China [14] Yuan/hm^2^.

Ecosystem services	Cropland	Forest	Grassland	Wetland
Gas regulation	23.35	1940.11	673.65	1082.33
Climate regulation	435.63	1827.84	700.60	6085.31
Hydrology adjustment	345.81	1836.82	682.63	6035.90
Erosion control	660.18	1805.38	1005.98	893.71
Waste treatment	624.25	772.45	592.81	6467.04
Biodiversity	458.08	2025.44	839.82	1657.18
Food	449.10	148.20	193.11	161.68
Raw materials	175.15	1338.32	161.68	107.78
Entertainment	76.35	934.13	390.72	2106.28

Total value	3247.90	12628.69	5241.00	24597.21

**Table 2:** Land use changes from 1980 to 2010 in the small Sanjiang plain.

Land use	Area (×10^4^ hm^2^)			Land use change (%)		
	1980	2000	2010	1980–2000	2000–2010	1980–2010
Cropland	262.19	346.62	434.82	32.20	25.45	65.84
Forest	222.57	215.18	182.83	−3.32	−15.03	−17.85
Grassland	24.62	23.12	2.09	−6.12	−90.96	−91.51
Wetland	171.74	95.63	56.29	−44.31	−41.14	−67.22
Building land	11.68	12.42	16.98	6.30	36.71	45.32
Wasteland	0.30	0.14	0.10	−53.17	−29.00	−66.75

**Table 3:** ESV change from 1980 to 2010 in the small Sanjiang plain.

	ESV (×10^8^ Yuan)			ESV change (×10^8^ Yuan)					
	1980	2000	2010	1980–2000	2000–2010	1980–2010
Cropland	85.16	112.58	141.23	27.42	28.65	56.07
Forest	281.07	271.75	230.89	−9.32	−40.86	−50.18
Grassland	12.91	12.12	1.10	−0.79	−11.02	−11.81
Wetland	422.43	235.24	138.47	−187.19	−96.77	−283.96

Total ESV	801.57	631.69	511.69	−169.88	−120.00	−289.88

**Table 4:** Single ecosystem service value change from 1980 to 2010.

Ecosystem services	Change (×10^8^ Yuan)		
	1980–2000	2000–2010	1980–2010
Gas regulation	−9.57	−11.75	−21.32
Climate regulation	−44.09	−27.48	−71.58
Hydrology adjustment	−44.48	−28.07	−72.55
Erosion control	−2.71	−5.65	−8.36
Waste treatment	−44.61	−23.68	−68.29
Biodiversity	−10.37	−10.80	−21.17
Food	2.42	2.44	4.86
Raw materials	−0.35	−3.55	−3.90
Entertainment	−16.13	−11.46	−27.59
